# Gender and age specific dynamics of health-related postoperative outcome measures following the laparoscopic sleeve gastrectomy

**DOI:** 10.1016/j.sopen.2024.10.007

**Published:** 2024-10-30

**Authors:** Sevak Shahbazyan, Mushegh Mirijanyan, Zhorzheta Badalova, Zareh Ter-Avetikyan

**Affiliations:** a Yerevan State Medical University, 2 Koryun Street, Yerevan, Armenia; b “Shengavit” Medical Center, 9 Manandyan Street, Yerevan, Armenia; c “Surgery” Medical Center, Nersisyan Street, Yerevan, Armenia

**Keywords:** Laparoscopic sleeve gastrectomy, BMI, BAROS, Age groups, Comorbidities, Complications, Body appearance concern

## Abstract

**Background:**

A variety of bariatric surgical techniques have been implemented to manage obesity, including the laparoscopic sleeve gastrectomy (LSG).

The aim of the study was to compare the pre and postoperative features of patients undergoing LSG, analyze the dynamics of BMI and BAROS indices and to assess the impact of body appearance concern on the postoperative outcomes.

**Methods:**

A total of 591 participants were divided into 3 age groups (20–39, 40–59 and over 60 years of age). The Charlson Comorbidity Index was used to assess comorbidity in patient groups. The rate of complications, assessment of psychological well-being and number of hospital days for patients were used to compare the features of postoperative recovery in different age groups.

**Results:**

In the young and middle-aged groups, the BMI reduction speed was similar, and significantly higher than in the elder group with a faster rate of BMI reduction in female participants. Higher scores of BAROS were revealed in the young and middle-aged groups for the 0–1 and 1–6 month periods. The 12-month assessment revealed much higher BAROS scores for male subgroups, compared to female participants. The BAROS scores decreased gradually in female groups within the assessment periods and was the lowest in the third assessment.

**Conclusions:**

The pattern of BAROS reduction can be the result of lower psychological and social well-being scores in female participants who reported significant weight reduction in 6–12 month period with paradoxically decreased quality of life scores explained by the impact of body appearance concern on the postoperative outcomes.

## Highlights

• Laparoscopic sleeve gastrectomy leads to BMI reduction in all age and gender groups.
• Health related quality of life (BAROS) was improved in male patients.
• BAROS data did not improve significantly in females despite the BMI reduction.
• Females had similar complication rate but lower psychological well-being scores.
• Effective stress management strategies can improve health related outcomes.

## Introduction

Obesity is a compound disorder derived from metabolic disturbances [1]. It imposes high risk of cardiovascular diseases, insulin resistance, atherogenic dyslipidemia, hypertension and many other disorders. The prevalence of obesity tends to increase correlating with the high rates of type 2 diabetes mellitus (type 2 DM) [2]. Obesity significantly increases the risk for early acute myocardial infarction, ischemic stroke and cardiovascular death [3]. According to the Global Health Observatory (GHO) about 2.8 million people die each year as a result of obesity. A number of international health organizations have ranked obesity as a separate disease [4]. The management options targeting obesity (dietary regulations, lifestyle modification, and drug interventions) unfortunately do not have proper efficiency and do not lead to reliable and irreversible improvement of the condition [5]. With the use of traditional management measures, not >10 % of patients with morbid obesity can achieve the desired treatment outcome [6]. Research evidence indicates that various weight loss programs, including diet therapy, drug therapy and physical exercises, can result in weight regain back to the obesity baseline [7,8]. The inefficacy of mentioned programs led to the development of other intervention approaches to treat obesity. Currently, the widely used surgical approach, bariatric surgery, is the only one that has been proven effective in reducing body weight in patients with severe obesity [9]. Variety of bariatric surgical techniques have been implemented to manage obesity, yet the laparoscopic sleeve gastrectomy (LSG) has currently become the most popular bariatric procedure worldwide due to its safety, effectiveness and technical simplicity [1,[9], [10], [11], [12], [13]].

The advantages of LSG became evident due to randomized clinical trial results [[14], [15], [16]]. The studies have compared different outcomes, safety, and the level of technical feasibility between the LSG and Roux-en-Y-gastric bypass procedure. Analysis of the long-term results obtained in patient groups that had undergone different bariatric surgery techniques is important to show the safety and substantial efficacy of the selected technique. Moreover, decreased rate of obesity-related comorbidity, increase in the quality of life and life expectancy indices are additional determinants of an effective intervention [17]. The current study provides the pattern of efficacy for the 12-month long follow-up of the postoperative outcomes of the LSG intervention with repetitive assessment of BMI, quality-of-life scores in patient subgroups.

Another factor influencing the health related quality of life in patients with high levels of obesity are the stress, shame, and anxiety of living with visible differences as documented in a comprehensive overview of the appearance and visible differences literature from a psychological perspective [18]. Obesity alters the individual's overall appearance, the way they perceive themselves, the way they are perceived by others and, therefore, impacts psychological and social well-being. In our work we have made a special focus on the analysis of these factors. Different authors [[19], [20], [21]] have assessed long term psychological functioning and outcomes in postbariatric patients, indicating persistence of anxiety in a small proportion of patients even in a 24-month follow-up period [22]. The low appearance self-esteem affects the psychophysical health and quality of life in obese individuals [23]. In our study we particularly tried to demonstrate the gender specific changes in psychological and social well-being of postoperative bariatric patients, with an effort to clarify the impact of the body-appearance concern on the psychological well-being. For that purpose we have used an Armenian version of specifically designed and validated questionnaire to address postoperatively gender specific measure of psychosocial adjustment to appearance concern in postbariatric patients, considering this as a main causative factor of psychological dysfunction.

The aim of the study was to compare the pre and postoperative features of patients undergoing LSG analyzing the dynamics of BMI and BAROS indices in different age groups of Armenian patients and to assess the impact of body appearance concern on the postoperative outcomes.

## Materials and methods

### Participants and the study design

This Prospective interventional single-clinic study with pre-post design was conducted in “Shengavit” medical center. Five hundred ninety-one patients who had undergone laparoscopic sleeve gastrectomy from March 2018 to March 2023 were enrolled in the study. The rationale for the convenience sampling design of the study was to ensure a complete (100 %) follow-up of the sample population of patients. A sample size was calculated using G*Power statistical analysis software considering an effect size of 0.85, alpha error probability of 0.05, power of 0.95, and allocation ratio was adjusted to the age structure of the population (N1(20–39 years) =287, N2(40–59 years) = 217 and N3(>60 years) = 86. N2/N1 = 0.75 and N3/N1 = 0.3) [24].

All patients recruited to participate in this study were informed about the risks and benefits of the LSG procedure. Informed consent was obtained from all the participants included in the study. The follow-up period of the study was 1 year.

The study protocol conforms to the ethical guidelines of the 1975 Declaration of Helsinki as reflected in the approval by human research committee. All procedures used in the study involving human participants were in accordance with the ethical standards of the Ministry of Health (MOH Republic of Armenia).

Patient inclusion criteria were as follows: age > 20, and body mass index in the following range: 40 < BMI < 55.

The exclusion criteria of the study were as follows: active *Helicobacter pylori* infection, non-treated peptic ulcer, previous gastric resection or fundoplication, drug or alcohol abuse, mental health disorders, age < 20, BMI < 40 and BMI > 55.

A total of 591 patients were selected to take part in the study. The participants were divided into 3 groups in accordance with their age. The younger group (*n* = 287) included the patients between 20 and 39 years of age, the middle-aged group (*n* = 217) were the patients between 40 and 59 years of age and the elder group (*n* = 86) composed of patients older than 60 years.

Thirty eight percent of participants involved in the study had comorbidities. In our study, the Charlson Comorbidity Index was used to assess comorbidity in groups. The index is based on the calculation of scores for the age and different comorbidities. To the sum of the scores for comorbidities, a point is added for every decade of life if the age of the patient is above 40 [25].

### Preoperative management and surgical intervention

The preoperative management of patients was performed by a multidisciplinary team. Medical, nutritional, endocrinological, and psychiatric standard preoperative assessments included abdominal ultrasound investigation, barium x-ray of the upper gastrointestinal tract with esophagogastroduodenoscopy, blood tests, cardiologic evaluation and chest x-ray. Psychiatric counselling was conducted to identify possible mental health contraindications to surgery [26]. Weight and dieting history, motivation for surgery and expectations concerning the operation outcomes were clarified in all participants.

### Assessment of the outcomes of bariatric surgery, psychological and social well-being

BAROS system has been applied by different research groups to assess QoL variables in the follow-up of bariatric patients. The BAROS tool contains four domains, targeting the percentage of excess weight loss, quality of life, changes in comorbidities, and post-surgical complications. A certain score is added for the first three domains, and the last domain score is reduced from the total sum. The responses from patients were evaluated according to Oria et al. [27] using the following grading of results: “insufficient,” “acceptable,” “good,” “very good,” and “excellent”.

For the assessment of physical and psychological distress and dysfunction level the Derriford Appearance Scale (DAS-59) was used [28]. Translation and adaptation of the scale had been conducted by Arakelyan et at all in an earlier study [29]. The DAS-59 generates six measures of psychological distress and dysfunction related to problems of adjustment and appearance.

The DAS-59 consists of a full-scale score and five factorial subscale scores: general self-consciousness of appearance (GSC), social self-consciousness of appearance (SSC), sexual and bodily self-consciousness of appearance (SBSC), negative self-concept (NSC) and facial self-consciousness of appearance (FSC).

Administration requires 10–15 min to complete and the scale is primarily intended for use by participants aged 16 years and older. It has a ‘non-applicable’ response category for most items to make the scale acceptable to all respondents. The DAS-59 consists of a series of 59 statements and questions with the answers categorized to measure the frequency of each symptom (Likert scale). These 59 items assess relevant physical and psychological distress and dysfunction. Higher scores on the DAS-59 indicate higher levels of distress and poorer levels of adjustment [28,30].

### Surgical intervention

In all patients the gastrectomy was performed laparoscopically by the same surgical team using four or five ports. LSG was performed with bougie size lower than 34F, and the gastric resection was carried out with a reinforced linear stapler (with three rows of small titanium staples) along the line of the bougie. The stomach tissue resected was removed from the abdominal cavity through one of the trocar holes.

### Postoperative management

All patients underwent early mobilization in postoperative 8–12 h. Upper gastrointestinal swallow test with Gastrografin was performed in all patients on the second postoperative day. Alimentary recommendations included a diet consisting of clear liquids and pureed foods for 15 days and a semisolid diet for the next 15 days. After 1 month, the patients gradually started to include in their nutritional plan a low-fat, low-carbohydrate and high-protein solid diet.

The main criteria used to compare the postoperative recovery in different age groups were the rate of complications measured by the Clavien-Dindo scale and number of hospital days for patients of three groups.

### Data analysis

Preoperative and postoperative BMI (body mass index), the rate of BMI reduction and results of assessment of BAROS were compared between the three clinical groups in one, six and twelve months following the operation. BMI calculation was performed in accordance with standard formula [[7], [8], [9]].

### Statistical analysis

Statistical data processing was performed using the statistical software package SPSS 23 (Statistical Package for Social Science 23) to determine any significant differences in post-operative scores between the groups. For a comparative analysis of the group results obtained before and after intervention the Kolmogorov-Smirnov test was used revealing the pattern of data distribution, followed by the Student's parametric tests for the comparison of group means. Chi square test was applied for the analysis of comorbidity proportions and rates of post-operative complications between the groups. The mean values for the length of hospital stay were compared using one-way analysis of variance (ANOVA). When using the Student test for independent samples, the calculation depended on the statistical significance of differences in the variance of the compared groups.

## Results

**Fig. 1:**
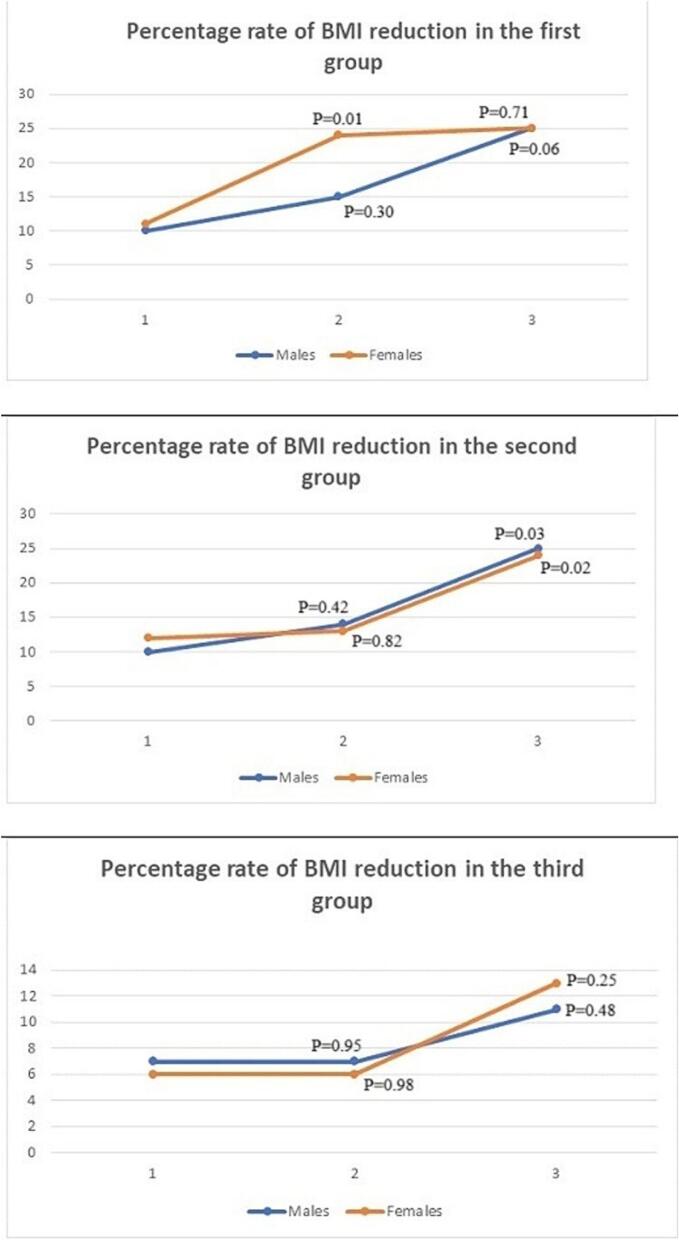
The speed or percentage rate of BMI reduction in all three age subgroups for male and female participants. The horizontal axis represents the three assessment timepoints (1 month vs initial level; 6 month vs 1 month and 12 months vs 6 months levels) and the vertical axis the percentage of reduction.

The study results were collected before the surgery, and compared with data from one, six and twelve-month follow-up assessments. Changes in BMI parameters of all groups were revealed in consecutive assessments following the surgery. The speed of BMI reduction for different age groups or percentage of reduction compared to the previous assessment are presented in Fig. 1.

Compared with the pre-operation data in all three age groups of the patients, changes in BMI indicators were already evident in a month after the intervention (Fig. 1). At the study baseline in patients of the group I, the initial levels of BMI were 46.3 ± 3.71 in males and 48.7 ± 4.2 in female patients. These values were reduced in a month following the operation (*P* < 0.05 and *P* < *0.05* respectively for male and female patients). In this age category reduction in BMI indicators was not remarkable between the one and six-month follow-up assessment in the men subgroup (*P* = 0.30) and significant reduction was persistent in the female subgroup (*P* = 0.01). The BMI reduction rate between six and twelve-month assessments was not significant (*P* = 0.06 and *P* = 0.71 respectively for male and female subgroups). In middle-aged groups BMI reduction rate was remarkable only at the third (six months vs twelve months) assessment (*P* = 0.02 and *P* = 0.03 respectively for male and female subgroups). For the elder groups the percentage reduction rate did not decrease significantly at second and third assessment periods (Fig. 1).

A specific difference was registered in the young age group (20–39), where the six-month assessment revealed a large amplitude of difference in the percentage rate of the BMI reduction between the male and female subgroups (14.7 % in males and 23.5 % in females).

All the patients were assessed for comorbidities to show the between group and between gender differences for the sample groups (Table 1).

**Table 1:** Comorbidities scores of three patient groups.

	Young group (age: 20–39 years)		Middle-aged group (age: 40–59 years)		Elder group (age: >60 years)		X^2^	P
	n = 287		*n* = 217		*n* = 86			
	Index score	Number and (%)	Index score	Number and (%)	Index score	Number and (%)		
The mean score of the Charlson Comorbidity Index	0–1	132 (53.6 %)	0–1	110 (51.4 %)	0–1	56 (51.9 %)	2.99	0.22
	2–3	87 (35.4 %)	2–3	78 (36.4 %)	2–3	34 (31.5 %)	1.61	0.45
	4–5	26 (10.6 %)	4–5	24 (11.3 %)	4–5	14 (12.9 %)	2.79	0.25
	≥6	1 (0.4 %)	≥6	2 (0.9 %)	≥6	4 (3.7 %)	10.13	0.006

A remarkable difference was revealed between one and six-month BAROS assessment means for the age category 1 (*P* < 0.05 and P < 0.05 in both gender subgroups) and the age category 2 (P < 0.05 and P < 0.05 respectively in male and female patients). While when comparing data for female patients from age category 2 between the 6 and 12 month time points, no significant difference in BAROS means was registered (*P* < 0.05 and *P* = 0.23 respectively in male and female patients). The results of BAROS between the one and six-month period assessments in the elder age category showed the same pattern of difference (*P* < 0.05 and *p* = 0.13), likewise the results showing the difference between the six and twelve-month periods in female subgroups were not remarkable (*P* < 0.05 and *p* = 0.25 respectively in male and female patients).

A significant difference was registered also for BAROS means in male patients of all the age categories at the twelve-month period assessment (P < 0.05).

To address the question regarding the comparatively decreased quality of life in the female subgroups, we have also conducted analysis of postoperative complications (Table 2). According to Table 2 the overall number of complications was relatively higher in female subgroups, yet the difference between the groups was statistically remarkable only for the 3b class (*P* = 0.02) showing significantly higher rate of complications in elderly female patients. The length of hospital stay data showed anticipated results with the length of stay increasing with age (Table 2).

**Table 2:** Main morbidity after the surgical intervention based on the Clavien-Dindo scale (%) and length of hospital stay for groups.

Young group (age: 20–39 years)			Middle-aged group (age: 40–59 years)			Elder group (age: >60 years)			X^2^ and P	X^2^ and P
Scale	Males	Females	Scale	Males	Females	Scale	Males	Females	Males	Females
	*n* = 158	*n* = 129		*n* = 103	*n* = 114		*n* = 44	*n* = 42		
1	9 (22.5 %)	13 (32.5 %)	1	8 (21.7 %)	11 (29.7 %)	1	3 (15 %)	4 (20 %)	X^2^ = 0.39 *P* = 0.82	X^2^ = 0.01 *P* = 0.99
2	4 (10 %)	7 (17.5 %)	2	4 (10.8 %)	6 (16.2 %)	2	1 (5 %)	4 (20 %)	X^2^ = 0.45 *P* = 0.79	X^2^ = 0.96 *P* = 0.62
3a	3 (7.5 %)	3 (7.5 %)	3a	2 (5.4 %)	4 (10.8 %)	3a	2 (10 %)	2 (10 %)	X^2^ = 1.08 *P* = 0.58	X^2^ = 0.65 *P* = 0.72
3b	–	1 (2.5 %)	3b	1 (2.7 %)	1 (2.7 %)	3b	1 (5 %)	3 (15 %)	X^2^ = 2.93 P = 0.23	X^2^ = 7.68 P = 0.02
4	–	–	4	–	–	4	–	–		
5	–	–	5	–	–	5	–	–		
Length of stay in days Mean ± SD	2.2 ± 1.2			3.4 ± 1.6			4.8 ± 1.8		ANOVA derived F and *P* values F = 118.6 P < 0.05	

The results of the psychosocial well-being assessment DAS 59 revealed higher scores for the female subgroups. Compared to the matched male subgroups significant difference was observed for some of the items included in the questionnaire.

## Discussion

Use of the bariatric surgery requires a proper analysis of the efficacy of the method and its impact on weight loss. Many studies have assessed the rate of weight loss for different bariatric techniques, including the LSG [[31], [32], [33]]. Different authors described the rate of weight loss following the LSG and have presented the average rates of long-term weight loss and the long term impact of LSG on the health related quality of life outcomes. However, there is lack of detailed data analysis in the literature presenting the age and gender-adjusted rate of weight loss following the LSG intervention and the dynamics of health related outcomes.

In our study we intended to evaluate the one month, six months and twelve-month results of BMI reduction in patients with 40 < BMI < 55 who were managed with LSG. The repetitive assessment of BMI and quality of life indices has revealed the dynamics of the weight improvement and its impact on the health related quality of life indices in the study participants.

Statistical assessment revealed faster and significant BMI reduction in female participants. Such pattern of weight reduction was observed in other studies describing the outcomes of different bariatric interventions for the 1–5 years of post-treatment period [34,35]. In the young and middle-aged groups the BMI reduction speed was similar, yet in the elder group BMI reduction rate was much lower. This difference is potentially explained by the lower rate of physical activity in the elder (age over 60) group, which has been shown in different systematic reviews [36,37].

The most common pattern of weight reduction was the initial higher speed of weight loss in all groups. The speed of reduction in all age groups was the highest in the first assessment period.

**Fig. 2:**
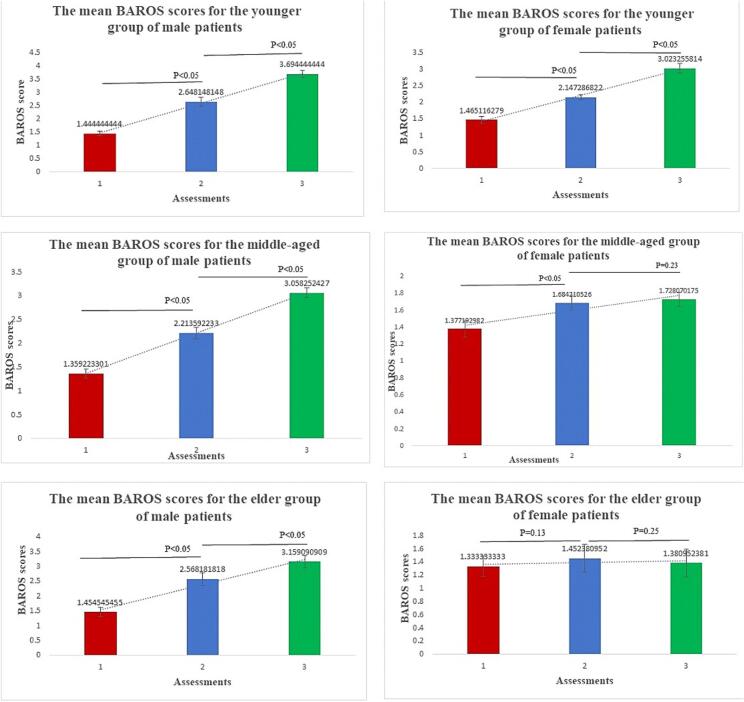
Comparison of gender specific BAROS scores (1-insufficient, 2-acceptable, 3-good, 4-very good and 5-excellent) for three age groups.

Another interesting aspect of outcome analysis was revealed when comparing the quality of life (BAROS) data (Fig. 2). Higher scores of BAROS were revealed in the young and in the middle-aged (40–60 years of age) groups, showing significant increase in the health related quality of life for the first and second assessment periods. This improvement was more evident for the male subgroups. The third assessment performed in twelve-month period revealed much lower BAROS scores for both subgroups, with remarkably reduced scores for the female participants. The quality of life index decreased gradually in female groups within the assessment periods and was the lowest in the third assessment. The Fig. 2 shows similar pattern of BAROS scale changes in male subgroups of all three age categories, with an ascending roofline pattern of the BAROS score distribution. In female subgroups this pattern was not observed, and higher amplitude of histogram was observed at the midpart of the data horizontal axis. This pattern of BAROS reduction in female participants contradicts the reported significant weight reduction in 6–12 month period. This paradoxical data in part might be explained by the impact of different confounding factors (gender specific and others) that potentially can deteriorate the scores of study outcomes. The analysis of confounding factors is necessary to show the net effect of the applied intervention even for six or twelve-month periods. The confounding includes factors like chronic and prolonged stress affecting negatively various aspects of life (physical, mental, and emotional health, interpersonal relationships, work and academic performance, and overall life satisfaction). In many patients not only the cardiovascular diseases, but also sleep disorders, anxiety, and depression are the result of excessive stress. The impact of stress and anxiety on the weight management process has been thoroughly studied by different research groups [38,39], yet the role of body appearance concern was not selectively addressed in these studies. The body appearance dissatisfaction is a strong candidate factor leading to stress and anxiety in overweight individuals [39]. Therefore the DAS 59 was used to assess the psychological and social well-being of the study participants before and after the surgery. As evident from Table 3 the DAS 59 scores had improved for all the subscales. There was no significant difference between the assessment scores at the baseline, yet the final assessment showed statistically significant improvement for majority of scales in all three subgroups. The differences between the final scores of the male and female subgroups were statistically significant for the full scale and subscale scores, demonstrating considerably better (lower) scores for the male subgroups in all assessment domains. Correspondingly, for some subscale assessments the difference between the pre- and postoperative assessment scores in female subgroups was not statistically significant (Table 3).

**Table 3:** Comparison of pre- and postoperative DAS-59 scores for age and gender subgroups.

Patient group	N	Full scale (SD)		DAS-59 subscales									
				GSC (SD)		SSC (SD)		SBSC (SD)		NSC (SD)		FSC (SD)	
		Pre	Post	Pre	Post	Pre	Post	Pre	Post	Pre	Post	Pre	Post
Male subgroup 1	108	90.5 (13.1)	62.3 (12.6)	34.9 (12.5)	23.9 (13.5)	26.2 (11.8)	16.7 (10.2)	10.4 (5.8)	5.5 (3.7)	17.8 (3.2)	14.4 (3.4)	4.1 (1.8)	1.9 (1.7)
Female subgroup 1	129	94.2 (16.3)	84.4 (16.8)	39.4 (15.4)	33.6 (16.8)	28.4 (14.4)	22.8 (12.6)	11.2 (6.3)	8.7 (4.3)	18.6 (3.6)	18.2^N^ (4.2)	4.4 (1.4)	4.2^N^ (1.6)
Difference (P value)		0.17	<0.01	0.16	<0.01	0.21	<0.01	0.31	<0.01	0.07	<0.01	0.15	<0.01
Male subgroup 2	103	92.6 (15.8)	72.4 (14.8)	36.3 (12.3)	27.5 (12.1)	27.6 (12.6)	19.8 (13.2)	10.8 (4.3)	7.8 (3.2)	18.3 (4.2)	16.8 (4.4)	4.2 (2.6)	2.2 (1.8)
Female subgroup 2	114	93.0 (17.2)	88.8^N^ (18.2)	36.8 (16.9)	34.2^N^ (18.2)	28.8 (15.2)	24.6 (10.4)	10.6 (5.8)	9.2 (3.7)	19.1 (4.3)	18.6^N^ (4.2)	4.6 (2.0)	4.0 (1.4)
Difference (P value)		0.86	<0.01	0.79	<0.01	0.51	<0.01	0.77	<0.01	0.15	<0.01	0.18	<0.01
Male subgroup 3	44	95.7 (19.9)	82.4 (16.4)	39.5 (14.7)	31.6 (12.1)	30.4 (12.4)	20.8 (12.9)	12.4 (4.8)	9.8 (4.6)	20.8 (5.3)	17.4 (4.7)	5.8 (2.8)	2.8 (2.3)
Female subgroup 3	42	96.8 (18.8)	90.4^N^ (17.8)	40.2 (18.7)	38.2^N^ (18.2)	33.8 (16.7)	27.2 (12.2)	13.7 (6.3)	12.2^N^ (3.7)	22.2 (5.9)	19.8^N^ (5.4)	6.4 (3.2)	4.2 (2.2)
Difference (P value)		0.79	0.03	0.85	0.05	0.29	0.02	0.28	<0.01	0.25	0.03	0.36	<0.01

The symbol N in some measurements indicates the statistically non-significant difference between the pre- and post-treatment assessment scores (*P* > 0.05).

According to different authors women are more likely than men to perceive themselves as obese or overweight and are more worried about their weight [40]. Hall KD at all demonstrated that compared to younger women, women over 40 years old were more likely to self-perceive themselves as overweight, to have ever dieted and to have lost at least 5 kg during their lifetime [41].

The body appearance concern was underlined by Glauert et al. as a core element of eating pathology and have been shown to be influenced by the visual environment, including appearance-related stimuli [42]. The authors have analyzed the impact of cognitive biases upon the appearance-related stimuli forming the body dissatisfaction. To our knowledge there are no any studies or data synthesis in the literature presenting the role of cognitive biases and their influence on the appearance-related stimuli in body dissatisfaction. This gap in the literature limits our understanding of the characteristics of these biases, and mechanisms of body dissatisfaction based on them. In this study we did not analyze the etiology of the body dissatisfaction and role of the cognitive biases in that domain.

A five-year longitudinal study assessing the psychological and social well-being of the postoperative patients can clearly map the long term scores of DAS 59 survey. The study can also clearly evidence the role of body appearance dissatisfaction and the etiology of the persisting concern. However, the follow-up of the bariatric patients for five years is challenging, as a large proportion of patients are from different countries or remote districts and majority of them will not re-attend the clinic in two years and the chance of future survey is further reduced.

It is essential to manage stress appropriately and adopt effective coping strategies to improve the quality of life. Promoting a healthy lifestyle and implementing effective strategies for the stress and body dissatisfaction management, particularly in female subgroups, can contribute significantly to the improvement of health related quality of life outcomes in patients who underwent bariatric surgery.

## Conclusion

The study results presenting the dynamics of BMI changes indicate that LSG performance in patients younger than 60 years of age is significantly more effective compared to the results registered in patients over 60. The data showed remarkable difference in results of BAROS questionnaire between the patient groups that were below and above 60 years of age as well as between the genders. Lower efficacy of LSG in patients older than 60 years of age is supposedly explained by higher rate of comorbidity and potentially lower level of physical activity. This paradoxical data of BAROS assessment in part might be explained by the possible impact of different confounding factors (gender specific and others) that potentially can deteriorate the scores of study outcomes. Paradoxically decreased quality of life scores are evidently explained by the impact of body appearance concern on the postoperative outcomes. Promoting a healthy lifestyle and implementing effective psychological management strategies addressing the body appearance concern may have remarkable impact on study outcomes.

## CRediT authorship contribution statement

**Sevak Shahbazyan:** Methodology, Investigation, Data curation. **Mushegh Mirijanyan:** Writing – original draft, Validation, Methodology. **Zhorzheta Badalova:** Writing – review & editing, Writing – original draft, Methodology, Investigation, Formal analysis. **Zareh Ter-Avetikyan:** Supervision, Methodology, Data curation, Conceptualization.

## Ethical approval statement

We received ethical clearance from the hospital research committee. Informed consent was obtained from all the participants included in the study.

## Funding

No Funding was received.

## Declaration of competing interest

The authors declare that they have no known competing financial interests or personal relationships that could have appeared to influence the work reported in this paper.
